# libxtc: an efficient library for reading XTC-compressed MD trajectory data

**DOI:** 10.1186/s13104-021-05536-5

**Published:** 2021-04-01

**Authors:** Nikolay A. Krylov, Roman G. Efremov

**Affiliations:** 1 Shemyakin-Ovchinnikov Institute of Bioorganic Chemistry, Russian Academy of Sciences, Miklukho-Maklaya st. 16/10, Moscow, 117997 Russian Federation; 2 Higher School of Economics, Myasnitskaya st. 20, Moscow, 101000 Russian Federation; 3 Research Center for Molecular Mechanisms of Aging and Age-related Diseases, Moscow Institute of Physics and Technology, Institutskiy per. 9, Dolgoprudny, 141701 Russian Federation

**Keywords:** Molecular dynamics, Biomolecular simulations, Parallel data processing, Efficiency of MD trajectories reading

## Abstract

**Objective:**

The purpose of this work is to optimize the processing of molecular dynamics (MD) trajectory data obtained for large biomolecular systems. Two popular software tools were chosen as the reference: the tng and the xdrfile libraries. Current implementation of tng algorithms and library is either fast or storage efficient and xdrfile is storage efficient but slow. Our aim was to combine speed and storage efficiency through the xdrfile’s code modification.

**Results:**

Here we present libxtc, a ready-to-use library for reading MD trajectory files in xtc format. The effectiveness of libxtc is demonstrated for several biomolecular systems of various sizes (~ 2 × 10^4^ to ~ 2 × 10^5^ atoms). In sequential mode, the performance of libxtc is up to 1.8 times higher and 1.4 times lower than xdrfile and tng, respectively. In parallel mode, libxtc is about 3 and 1.3 times faster than xdrfile and tng. At the same time, MD data stored in the xtc format require about 1.3 times less disk space than those treated with the tng algorithm in the fastest reading mode, which is a noticeable saving especially when the MD trajectory is long and the number of atoms is large—this applies to most biologically relevant systems.

**Supplementary Information:**

The online version contains supplementary material available at 10.1186/s13104-021-05536-5.

## Introduction

Molecular dynamics (MD) is one of the most powerful and widely used methods for atomistic modeling of biological systems. It is often employed to study such phenomena as protein folding, assembly and binding to cell membranes, protein–protein and protein–ligand interactions, and many others (see [1] for recent review). The results of the MD experiment are stored in the so-called MD trajectory file, which contains information about the coordinates and (if necessary) velocities of each atom in every defined moment of time. Together with information about the molecular topology, this creates a huge array of raw MD data, that require further processing.

Although many libraries and ready-to-use applications for MD data processing exist, e.g.: MMTK [2], CPPTRAJ [3], HTMD [4], LOOS/PyLOOS [5], MDAnalysis [6], MDTraj [7], Pteros [8, 9], VMD [10], ST-Analyzer [11], GROMACS [12], there is, in our opinion, a prominent need today for mid-level tools for large-scale MD data analysis, which allow users with basic programming skills to adapt existing code to new tasks relatively easy and quickly. This need was further stimulated by the necessity for straightforward and flexible implementation of our original techniques of biomolecular simulations and analysis methods, accumulated over more than 20 years of work in this field (see some of the relevant reviews: [13–15]) and resulted in the development of the integrated framework for simulations and computational analysis of complex molecular ensembles. The MD trajectory processing is a core part of the framework.

Despite the existence of the modern tng storage format and the optimized library handling tng input and output (I/O) operations [16], the older xtc format and the xdrfile library (a standalone part of the GROMACS package for reading and writing trr and xtc files) are still commonly used.

In this work, we propose a ready-to-use libxtc library for reading MD trajectory files written in xtc format which is a part of our in-house integrated framework. The efficiency of libxtc is demonstrated for several biomolecular systems of different size.

## Main text

### Methods

Preliminary performance measurements have shown that the xtc frame reading algorithm [17] is primarily compute-bound. Therefore multi-threaded parallelization can be used to increase read speed employing the proper workload distribution scheme. The scheme implemented in libxtc is simple: each thread reads the control bits from the input file and, depending on the thread index and iteration counter of the main loop, either unpacks the current data block or skips several consecutive data blocks starting from the current one (see Additional file 1: Algorithm S1). Hence, the full decompression time is reduced when using more than one central processing unit (CPU), since the control bits reading time is small compared to the time of unpacking the data block. The number of atomic coordinates in a single data block (batch size) is adjusted at runtime to keep the workload disbalance between threads below ~ 20% for small systems (< 10^4^ atoms), while the batch size is fixed for larger ones as it has little effect on read rate when the number of atoms is greater than ~ 10^5^ according to performance measurements. Furthermore, to take advantage of modern CPU capabilities, an intermediate 64-bit buffer was added allowing aligned memory access and making bit reading from memory faster. To speed up inner loops, 64- and 128-bit integer arithmetic operations were used, if they were supported by the compiler and the target CPU. These techniques are similar to those applied in the tng library [16], but were developed independently.

The libxtc library implements frame search and skip algorithm intended for the trajectory file containing frames with the monotonically increasing time.

### Results

To test the optimization techniques implemented in the library, we compared the performance of libxtc, xdrfile and tng using MD trajectories obtained for the molecular systems enumerated in Additional file 1: Table S1. The test systems were selected based on the following criteria. First, they represent real cases that are commonly considered in biomolecular simulations. Second, they have significantly different sizes, and still contain at least 1.5–2 × 10^4^ atoms. For smaller systems, overall processing time is typically not too sensitive to the trajectory formats and compression algorithms.

Performance measurements were carried out using the hardware and software components listed in Additional file 1: Table S2. The results obtained are shown in Additional file 1: Table S3 and Figure S1. To collect the results and estimate the steady-state reading speed, 1000 frames were read 20 times for each format. To calculate the average reading speed and its standard deviation, the values of the reading speed from each run were used. The same procedure was applied to evaluate xtc reading speedup in multithreaded mode.

A tng-compressed trajectory has single frame in each frame set, otherwise the performance of the algorithm drops proportionally to the number of frames in the set. Inter-frame compression gives the output file ~ 20–30% smaller than xtc-compressed, but this gain only appears when the output time step is less than 10 fs, which makes inter-frame compression impractical.

The second set of tests is aimed at measuring the dependence of the performance of libxtc on the number of CPUs (Additional file 1: Figure S1). To estimate the acceleration, only the decompression time was taken into account due to the aforementioned observation that measurement results are significantly influenced by the hardware. The tng and xdrfile acceleration data are not shown, due to the single threaded structure of their code.

### Discussion

In all cases libxtc performance was about 1.5–1.8 times higher than xdrfile, but c.a. 1.4 times lower than tng. However, additional tests with other hardware have demonstrated that the read rate varies considerably depending on the hardware running the test code, and both the read rate values for each algorithm and the pairwise relative performance of the algorithms deviate from the data in Additional file 1: Table S3. It is not possible to test every possible hardware setup, but the performance data obtained for an additional modern configuration is shown in Additional file 1: Table S4 to illustrate the range of possible performance values. Along the way, it can be noted that an observable increase in the decompression rate of xtc frames was achieved with a fairly simple optimizations and code restructuring.

Additional file 1: Figure S1 shows that the slope of the acceleration coefficient curve for 1–4 CPUs is about 0.5, which indicates that the performance gain is not ideal. In addition, the speedup saturates when using more than 4 CPUs. This is rather expected because the time spent by each CPU while reading input data control bits and the time required to begin and end parallel code execution do not decrease when using more processors. This irreducible overhead leads to a faster saturation of the acceleration gain for small systems. Still, the maximal achieved read rate is comparable to that of tng. It was ~ 600 frames per second for the medium system and ~ 350 frames per second for the large one (according to the separate set of test runs), so the proposed algorithm meets the stated requirements. However, there should be some space to improve it even in single-threaded mode—this is clearly illustrated by the performance of the tng file I/O library.

Additional file 1: Table S3 shows that the overall improvement in storage format and compression algorithms has its cost in terms of storage requirements per atom. For the largest system with a write interval of 100 picoseconds, a tng-compressed file consumes about 30% more storage space than a compressed xtc one. In other words, one has to consider a compromise: (i) faster compression/decompression, but a larger file size for tng; or (ii) smaller files, but a lower read rate for xtc, when using existing software.

## Conclusion

The libxtc library, that combines processing speed of the modified decompression algorithm with storage efficiency of the old but widely used xtc file format, can accelerate analysis of long MD trajectories containing lots of atoms—typical for many currently studied biological system models. The libxtc is meant to be used as a standalone package or be integrated into the existing MD analysis software.

## Limitations

Optimizations implemented in libxtc either reduce or eliminate the performance gap between the xtc and tng formats when the number of atoms in the MD system is large (but less then ~ 10^8^ particles, due to xtc file format limitation) and the recording interval is greater than 5–10 fs, due to reduction of the interframe compression algorithm effectiveness, mentioned above. It is important to note that most of biologically relevant systems—proteins, biomembranes, and so on—meet the stated criteria. Therefore, libxtc seems to be promising for the processing of MD results obtained for this wide class of biological objects.

Regardless of the storage format used, the difference in trajectory processing speed will be more pronounced for tasks that require a comparable amount of time to read the trajectory and perform required calculations.

## Supplementary Information

**Additional file 1.** Supplementary Materials. This file contains data (pseudo-code, tables, figure) referenced in the text.

## Data Availability

The datasets analysed during the current study are available from the corresponding author on reasonable request.

## Abbreviations

CPU: Central processing unit
fs: Femtosecond
I/O: Input and output
MD: Molecular dynamics

## Availability and requirements

Project name: libxtc.

Project home page: https://gitlab.com/impulse_md/libxtc.

Operating systems: All CPython-supported OS. The pre-built binary packages for Linux, Windows, macOS (x86 or amd64 CPU).

Programming language: Python, C++.

Other requirements: numpy.

License: GNU GPL v3.
