# Supplementary material for: libxtc: an efficient library for reading XTC-compressed MD trajectory data
Source: BMC Res Notes. 2021 Apr 1;14:124. doi: 10.1186/s13104-021-05536-5 (PMC8017739; doi:10.1186/s13104-021-05536-5)
Supplement: Supplementary file 1 — Additional file 1. Supplementary Materials. This file contains data (pseudo-code, tables, figure) referenced in the text. [file 13104_2021_5536_MOESM1_ESM.pdf]

libxtc: An efficient library for reading XTC-compressed MD trajectory data.

Nikolay A. Krylov, Roman G. Efremov

Algorithm S1: Parallel xtc frame decompression algorithm.

```
tid - current thread index
nt - total thread count
natoms - number of atoms in the current frame
refcntr ← 0
batch_size ← max(1, min(512, natoms/(5·nt)))
while has more data do
   skip ← (refcntr/batch_size + tid) mod nt
   refcntr ← refcntr + 1
   if skip ≠ 0 then
      skip coordinate bits
   else
      unpack coordinate bits
   end if
   rf ← extract rle flag
   if rf ≠ 0 then
      run ← extract run length
      if run ≥ 0 then
         if skip ≠ 0 then
            skip rle bits
         else
            unpack rle bits
         end if
      end if
   end if
end while
```

Table S1: Size and composition of molecular systems used for testing.

System type	Size	Components	Description
Small	20138	1 protein and 6649 water molecules	19-residue peptide in a water box
Medium	118812	288 dioleoylphosphatidylcholine (DOPC) molecules (54 atoms each) and 34420 water molecules	Hydrated lipid bilayer
Big	210566	4 protein chains (4859 atoms each), 257 palmitoyloleoylphosphatidylcholine (POPC) molecules (134 atoms each), 119 palmitoyloleoylphosphatidylethanolamine (POPE) molecules (125 atoms each), 150 cholesterol molecules (74 atoms each), 43558 water molecules 1 Ca^{2+} and 42 Cl^{-} ions	Tetrameric integral membrane protein (receptor TRPV1) in three-component hydrated lipid membrane

Table S2: Hardware and software components used for testing.

	Configuration 1	Configuration 2
CPU	Intel Xeon CPU E5-2667 v2	AMD Ryzen 7 3700X
Memory	DDR4 1866 MHz	DDR4 3200 MHz
Chipset	Intel C602J	AMD X470
Storage	HDD: HGST HUS726040ALE614, SATA III	SDD: Samsung 970 EVO Plus 500GB, M.2, PCI-E 3.0 4x
Operating System	Fedora Core 30	Gentoo
Compiler	gcc 9.2.1	gcc 9.3.0

Table S3: Performance of data processing and storage requirements for various molecular dynamics (MD) trajectory file formats and libraries libxtc, xdrfile, tng in the single-threaded mode.

	Data processing speed[a]			Required storage[b]	
System type	libxtc [c]	xdrfile	tng	xtc	tng
Small	1711±8	1110±20	2460±130	71.2 (3.62)	71.9 (3.65)
Medium	327±0.5	185±3	450±13	431 (3.72)	575 (4.96)
Big	187±1	105±2	260±7	762 (3.71)	1015 (4.94)

a) The values are given in MD frames per second (fps). 1000 frames of MD production run with a recording interval of more than 10 ps were used to estimate fps.
b) Average disk space required per single MD trajectory frame in kilobytes for the XTC and TNG formats (in brackets - data in bytes per atom).
c) This work.

Table S4: Performance of data processing on the second hardware configuration in the single-threaded mode. Other details are identical to the ones of Table S3.

	Data processing speed		
System type	libxtc	xdrfile	tng
Small	4040±6	1540±10	6300±100
Medium	697±1	256±2	4540±70
Big	402±0.5	143±3	930±10

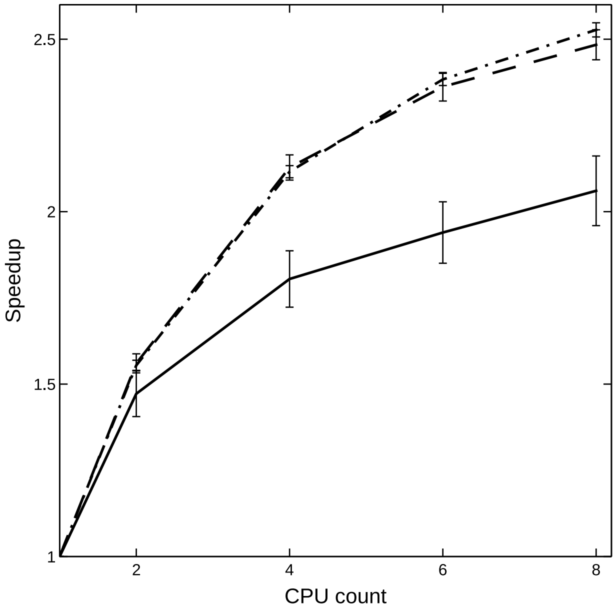

Figure S1: CPU-dependent acceleration of xtc frame reading for small (solid), medium (dash), and large (dash-dot) test systems. X axis is the number of used CPUs, Y axis is the speedup (coefficient of acceleration). Value deviations shown as vertical bars.

Abbreviations

CPU: Central processing unit

fps: Frames per second

MD: Molecular dynamics

MHz: Megahertz

rle: Run-length encoding
